# Supplementary material for: Systematic analysis of protein turnover in primary cells
Source: Nat Commun. 2018 Feb 15;9:689. doi: 10.1038/s41467-018-03106-1 (PMC5814408; doi:10.1038/s41467-018-03106-1)
Supplement: Supplementary file 1 — Supplementary Information [file 41467_2018_3106_MOESM1_ESM.pdf]

Systematic analysis of protein turnover in primary cells

Mathieson et al

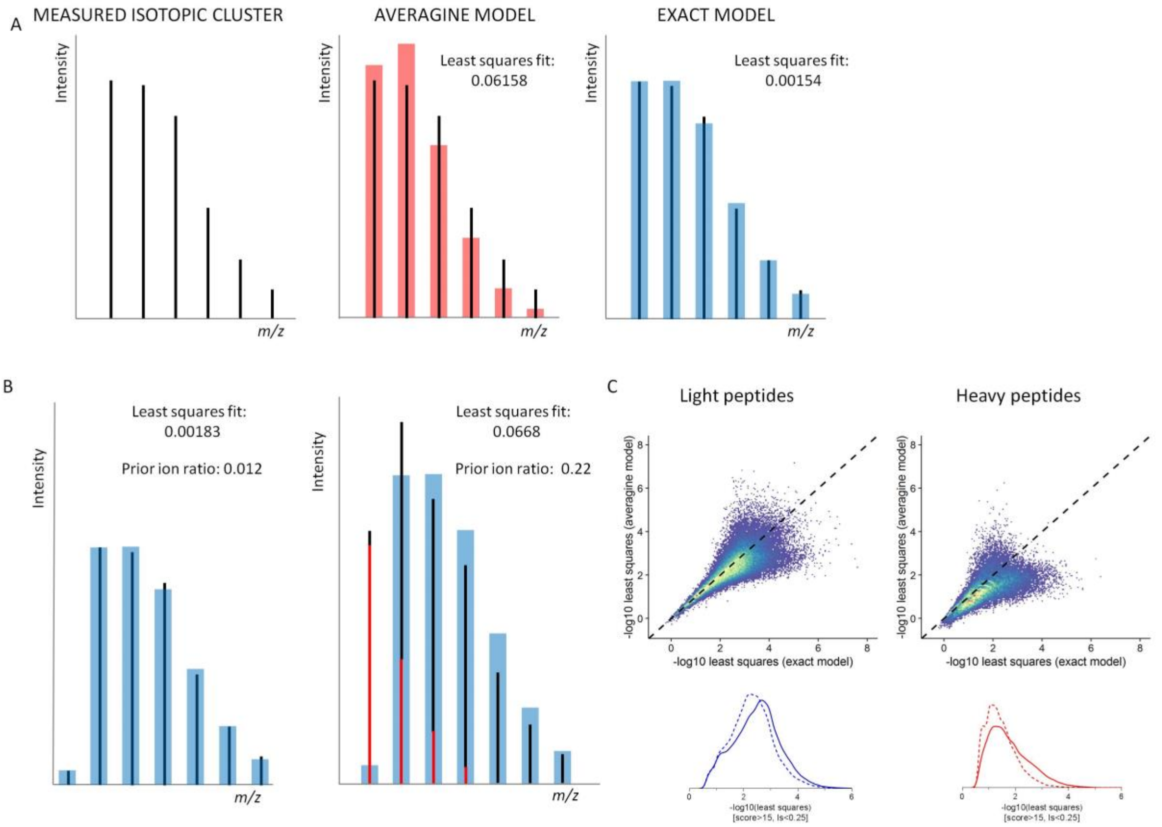

Supplementary Figure 1: Least squares fitting of the theoretical envelope to acquired isotopic clusters and calculation of 'prior ion' ratio. **A** Example of fitting the averagine model, here depicted as red bars, to a measured isotopic cluster. Following identification of a peptide and modification(s) by the Mascot search engine it is possible to construct an exact model from its elemental composition. An intensity for the heavy or light peptide is calculated by fitting the corresponding exact model of the peptide (here the SILAC-light form of peptide GPCMSEQAMGPCMSEQAMK) to the acquired data using a least-squares method **B** In this example, the theoretical isotopic envelope of the SILAC-heavy form of the peptide GPCMSEQAMGPCMSEQAMK is fitted to the acquired intensities. A low intensity, 'prior ion' is observable prior to the intense monoisotopic peak. The ratio of the sum of the intensities fitted to the theoretical envelope by least squares fitting (blue bars) divided by prior ion intensity is termed the 'prior ion ratio'. In the first case this ratio is small because no overlapping isotope cluster is present (black sticks), but in the second, an indistinguishable interfering / co-eluting cluster (red sticks) is shown which results in a high prior ion ratio despite a good fit via least squares. **C** The exact model fits better to the experimental isotopic envelopes. Data from thirteen raw files originating from one cell type (monocytes) harvested at a single time point (7 hours after swapping to heavy medium) analysed with isobarQuant, once with an exact model and then with an averagine model. Upper panel, left: Scatter plot of -log10 transformed least squares fits of the same light peptides (Mascot score >15) for the exact model (x-axis) versus the

averagine model (y-axis). Right: as previous but for the heavy SILAC peptides. Lower panel: density distribution of the –log10 transformed least squares fits, where least squares fit is less than 0.25. The dashed line represents the averagine model and the solid line the exact model. Left, light peptides; right, heavy peptides.

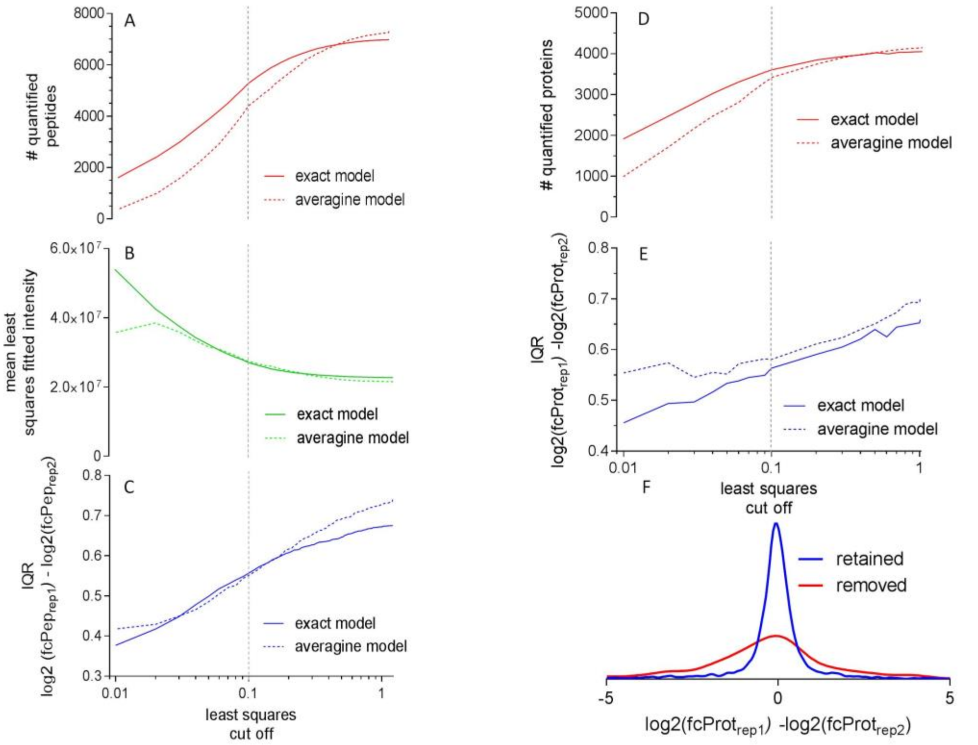

Supplementary Figure 2: Assessing the impact of the least squares fits [for exact and averagine model] of labeled peptides contributing to protein quantification, mean fitted intensity and peptide fold change reproducibility. Data from thirteen raw files originating from one cell type (monocytes) harvested at a single time point (7 hours after swapping to heavy medium) analysed with isobarQuant, once with an exact model and then with an averagine model.

The gray dashed line shows the selected threshold: 0.1 for the quality of the least squares fits of peptide isotopic distributions calculated as the sum of the squares of the residual values **A** The number of quantified peptides decreases slowly until approximately the 0.1 cutoff after which it drops faster. While the exact model starts with around 300 (around 5%) fewer quantified peptides than the averagine model these are filtered away at the cutoff around 0.5 least squares. At all cutoffs after this point there are consistently considerably more peptides from the exact model. **B** The mean least squares fitted intensity increases as more stringent cutoffs are applied, the trend is more pronounced for the exact model than the averagine model. **C** Reproducibility of quantification improves with more stringent filters. A substantial improvement compared to all peptides is achieved when applying the filter at 0.1, with low loss of peptides. While the averagine model at the 0.1 cutoff yields the same reproducibility as the exact model, the peptide losses for the averagine model at this cutoff are much higher. **D** On the level of protein fold change the number of quantifiable proteins decreases with increasingly stringent least squares cutoffs, slowly up to the least squares cutoff at 0.1, where 87% of all proteins are still

quantifiable. After this point the rate at which proteins are filtered with each least squares cutoff increases. **E** A substantial improvement in reproducibility (measured by the interquartile range of the delta log_2 fold change between replicates) compared to all proteins is achieved using the exact model when applying the filter at 0.1, with relatively low loss of proteins. A similar trend is observed for the averagine model up to a least squares cutoff of 0.1, however the protein fold changes are less reproducible throughout; after this cutoff the reproducibility for the averagine model becomes more unstable. **F** Improved precision in protein quantification as a result of least square fit based filtering using the exact model. Density distributions of protein log_2 fold change difference between two biological replicates for proteins quantified using peptides with least squares fit below or equal to 0.1 (blue line), standard deviation, σ=1.21 and with peptides with least squares fit above 0.1 (red line), standard deviation, σ=2.15.

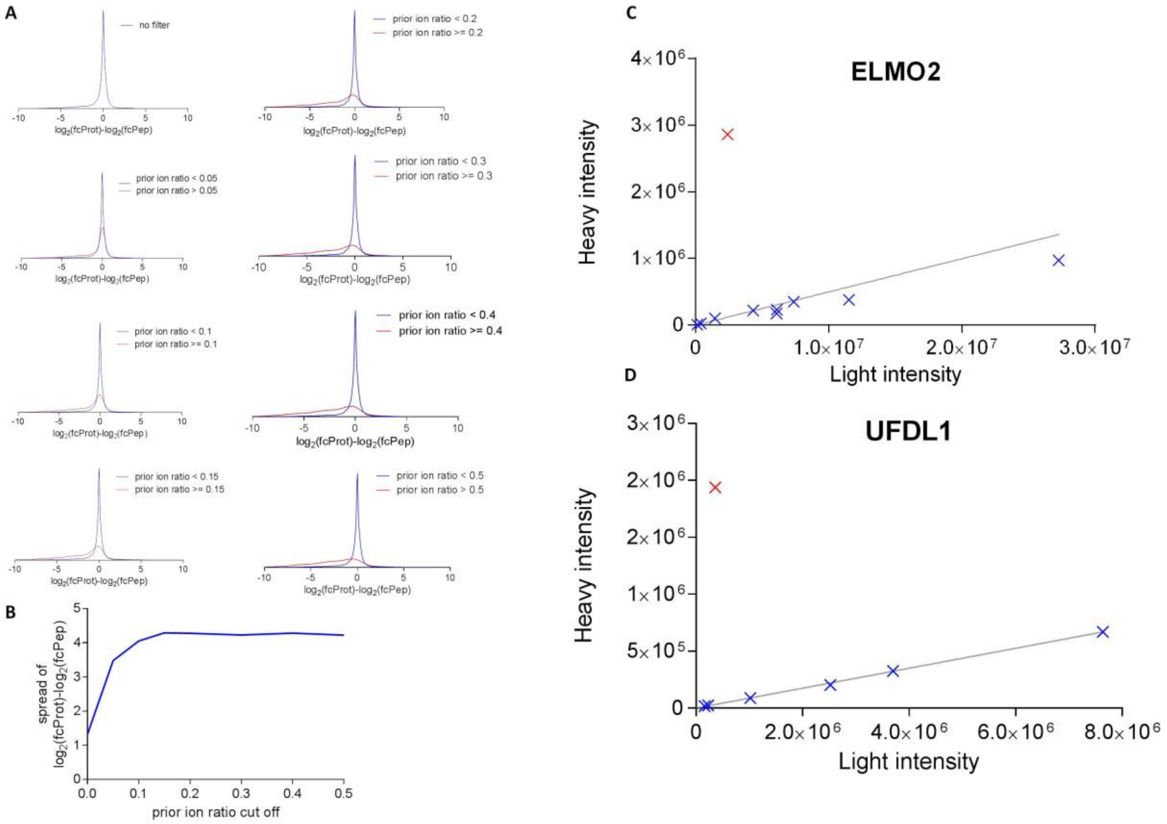

Supplementary Figure 3: Effect of prior ion ratio on fold change accuracies. Data from thirteen raw files originating from one cell type (monocytes) harvested at a single time point (7 hours after swapping to heavy medium) analysed with isobarQuant, once with an exact model and then with an averagine model. **A** Density plots of the spread of peptide fold-change deviations (determined by subtracting the log_2 fold change ratio of the peptide from the log_2 of the protein fold change [median]) for data retained or filtered out at increasingly stringent cutoffs. The plot is skewed for data filtered away at cutoffs greater than 0.15, indicating that there is a consistent overestimation of peptide fold changes in these data. At a prior ion ratio cutoff of 0.15 and below the spread of the data is narrow, at higher cutoffs the spread is substantially wider. A plot of the quartile ranges of the spread of accuracy data **B** confirms that the spread is greater in data with a prior ion ratio of greater than 0.15. **C & D** Examples of proteins with inaccurate peptide fold change ratios indicated by a high prior ion ratio value. The slope of the line given in gray represents the fold change as determined by the median of all valid peptide fold changes for that protein. **C** For ELMO2 the single outlying peptide (red cross) has a fold change of 1.2 (compared to the median fold change of 0.05) and a prior ion ratio of 3.002, with all other peptides having a prior ion below 0.2 (blue crosses). **D** For UFDL1 the outlying peptide (red cross) has a fold change of 5.4 compared to the median fold change of 0.09 for the protein. It has a prior ion ratio of 1.194 which contrasts to all other peptides (blue crosses) which have a prior ion ratio below 0.2.

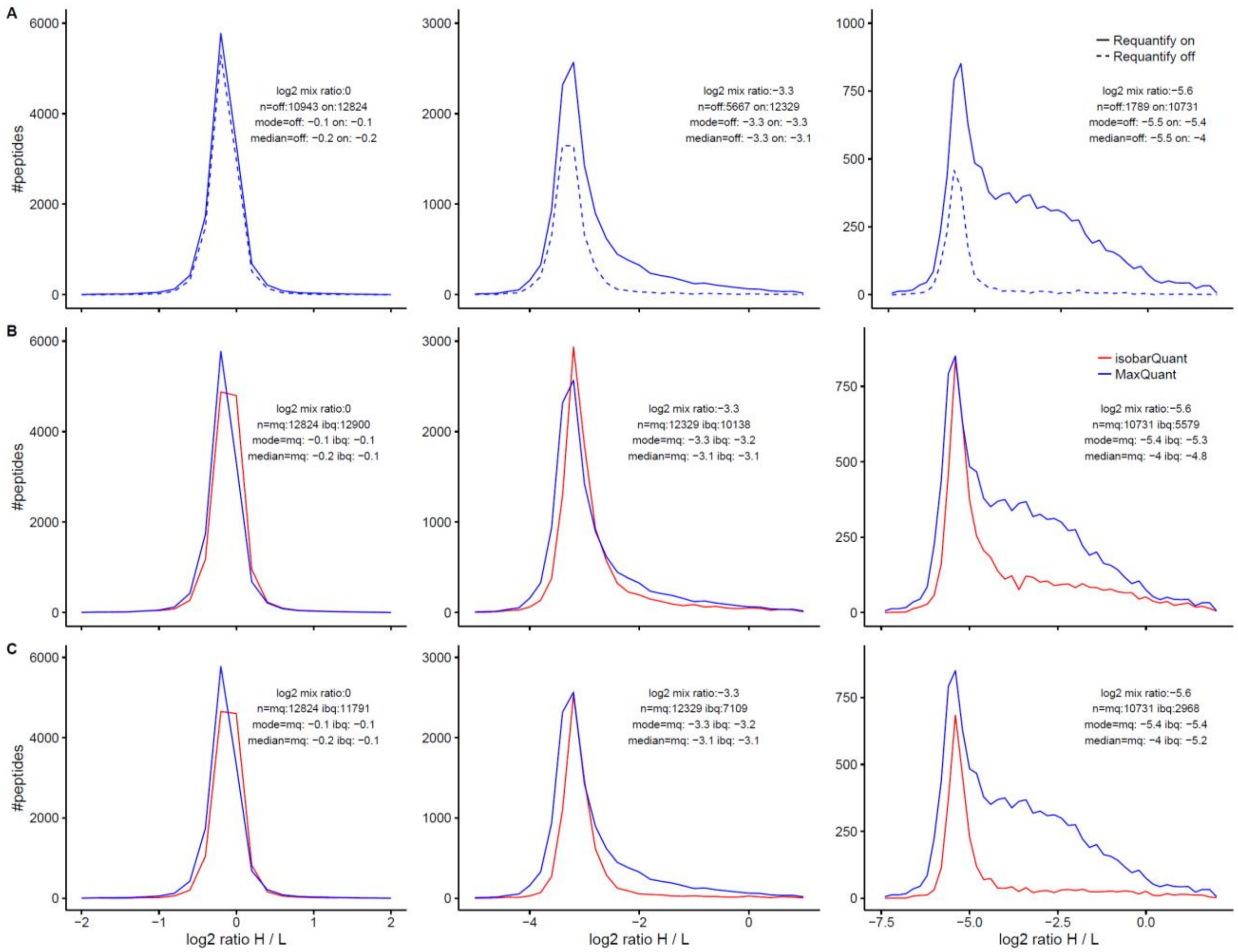

Supplementary Figure 4: Assessment of peptide and protein ratios obtained using isobarQuant and MaxQuant for three dilutions of heavy SILAC. We mixed light and heavy SILAC labeled THP1 cells at different ratios, and analysed the lysed and digested sample without any pre-fractionation thus creating a particularly demanding task for accurate quantification. From left to right decreasing amounts of heavy SILAC are mixed with light SILAC medium (column 1: 1:1 (0 $\log_2$ ratio), column 2: 1:9 (3.2 $\log_2$ ratio) and column 3: 1:49 (5.6 $\log_2$ ratio)). The deviation in accuracy of the mode value (the most frequently occurring value in the distribution) for the 1:1 mix is representative for the deviation due to pipetting precision. We compared the performance of isobarQuant to MaxQuant using both the widely used and recommended setting of re-quantify “on” as well as re-quantify setting turned off. Re-quantify attempts to rescue and find new peptide signals by looking in the relevant retention time window for peaks that would fit into the expected isotopic pattern. This function significantly increases the number of accurately quantified peptides, but comes at a cost of including a high amount of less well quantified ones. The isobarQuant strategy for going back and quantifying peptides after peptide identification, is conceptually similar to MaxQuant’s re-quantify function. This strategy evaluates the quality of each theoretical and experimental isotope cluster match and is able to extract the accurately quantify peptides significantly better than MaxQuant when large ratios are measured, which is apparent from the more accurate median value for the 1:49 sample, the addition of filter criteria based on prior ion and isotopic fit quality filters away the poorly quantified peptides and further improves the median value.

A Comparison of the quantification performance on the peptide level when using the MaxQuant re-quantify function (solid line), and when this function is turned off (dashed line). Without the re-quantify function MaxQuant is able to retrieve accurate quantification values but there is a significant drop in quantified peptides. While this reduces the number of inaccurately quantified peptides, a substantial portion of accurately quantified peptides is also lost.

B IsobarQuant without the filtering step retrieves as many accurately quantified peptides as MaxQuant but significantly fewer poorly quantified ones. This is immediately clear from the median values of the 1:49 sample (-4 for MaxQuant and – 4.8 for isobarQuant).

C IsobarQuant with the filtering steps used in this study (means least squares fit < 0.1 and prior ion ratio < 0.2) retrieves a large number of accurately quantified peptides and manages to discriminate and exclude the poorly quantified peptides, which is reflected in a median value of -5.2 in the 1:49 sample.

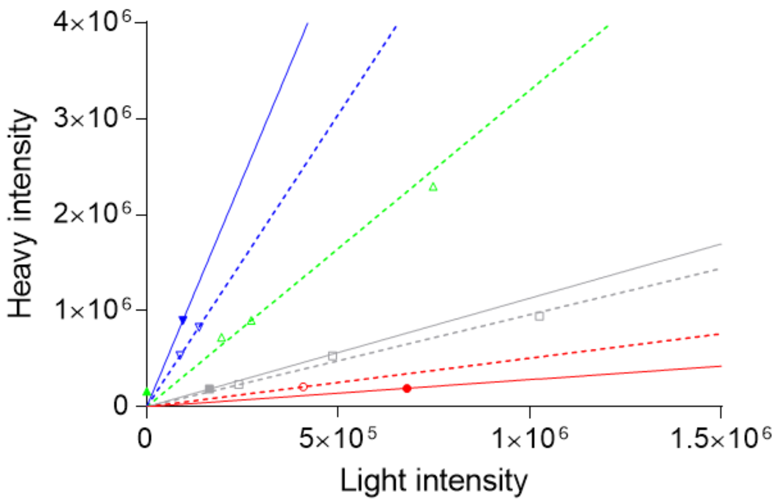

Supplementary Figure 5: Fold changes of JAK3 in B-cells. JAK3 (P52333) has a short half-life of 9.25 hours in replicate one and 11.65 in replicate two. The underlying peptide ratios are shown for each time point in each replicate and are represented by different colors (red, 7 hours; gray 11 hours; green 24 hours and blue 34 hours). The protein fold changes are shown by the solid (replicate one) or dashed lines (replicate two).

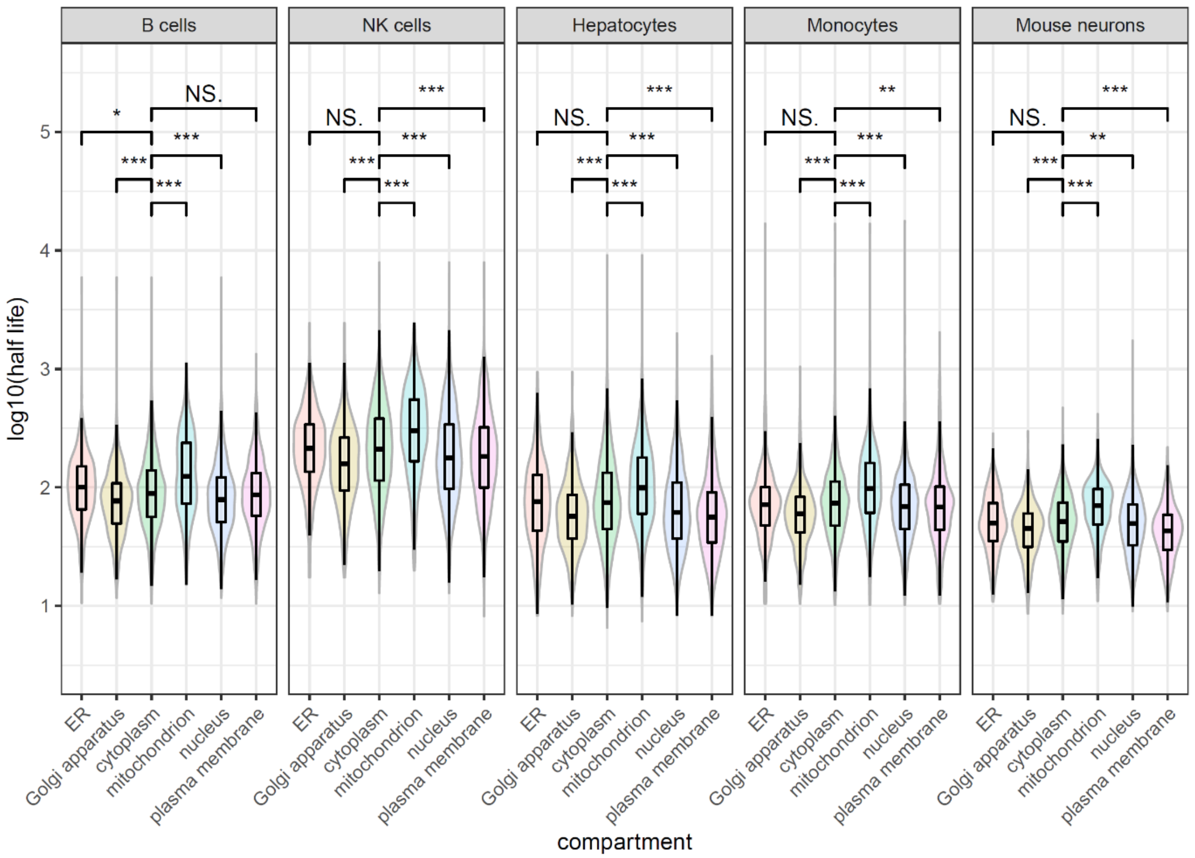

Supplementary Figure 6: Protein turnover in different cellular compartments. Distribution of protein half-lives in different cellular compartments. All proteins annotated for the GO terms endoplasmic reticulum (GO:0005783), Golgi apparatus (GO:0005794), cytoplasm (GO:0005737), mitochondrion (GO:0005739) and nucleus (GO:0005634) were retrieved using the R package AnnotationDbi. For each cell type, measured proteins were matched to these GO terms. If a protein was annotated for several compartments, it was assigned to all of these compartments. Distributions of log10 transformed half-lives were plotted for each compartment group in each of the cell type with the R package ggplot2 and significance was assessed with the package ggsignif using Wilcoxon-rank test (significance levels were encoded as ***: $p < 0.001$, **: $p<0.01$, *: $p<0.05$). Centre line in box plots is the median, the bounds of the boxes are the 75% and 25% percentiles i.e. the interquartile range (IQR) and the whiskers correspond to the highest or lowest respective value or if the lowest or highest value is an outlier (greater than 1.5 * IQR from the bounds of the boxes) it is exactly 1.5 * IQR.

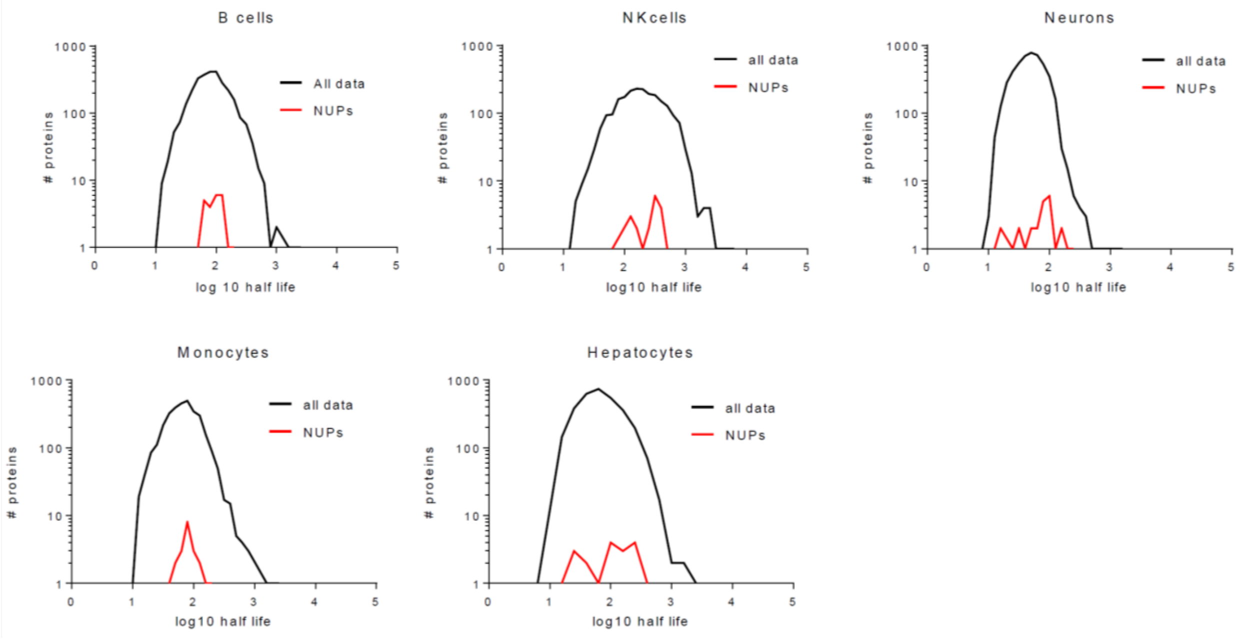

Supplementary Figure 7: Distribution of half-lives of NUPs identified in each cell type. The distribution of the log10 transformed protein half-lives was plotted for all proteins and specifically for the Nups. In all cases the turn over (expressed as log10 half-life) for these proteins is located towards the middle of the range for all proteins.
