# Supplementary material for: Systematic analysis of protein turnover in primary cells
Source: Nat Commun. 2018 Feb 15;9:689. doi: 10.1038/s41467-018-03106-1 (PMC5814408; doi:10.1038/s41467-018-03106-1)
Supplement: Supplementary file 3 — Description of Additional Supplementary Files [file 41467_2018_3106_MOESM3_ESM.pdf]

Description of Additional Supplementary Files

File Name: Supplementary Data 1
Description: Summary of time points used for dynamic SILAC labelling and raw file names.

File Name: Supplementary Data 2
Description: Protein half-lives from all cell types.

File Name: Supplementary Data 3
Description: Protein half-lives proteasome.

File Name: Supplementary Data 4
Description: Protein half-lives nuclear pore.

File Name: Supplementary Data 5
Description: MaxQuant settings.
